# Mental Capacity Legislation edited by Rebecca Jacob, James Gunn, and Anthony Holland, Second Edition, RCPsych/Cambridge University Press, June 2019, Hardback, ISBN 9781108480369, £29.99 — CORRIGENDUM

**DOI:** 10.1192/bjb.2020.11

**Published:** 2020-08

**Authors:** Martin Curtice

**Affiliations:** Coventry and Warwickshire Partnership NHS Trust, Warwick, UK. Email: mjrc68@doctors.org.uk

This second edition book reviews significant changes to the Mental Capacity Act (MCA) and readily accomplishes its aim to help clinicians in their daily practice. It is a short book of seven chapters and 120 pages and is written in an engaging style, being expertly presented with the use of sub-headings and crafted case studies, and avoiding large tracts of boring text.

Although all chapters are pertinent, the chapters on assessment of capacity and best interests stand out. Sage advice and clarity in breaking down the constituents of the capacity assessment elucidate the intricacies of assessing capacity that can be taken for granted at times in the hurly burly of clinical work. The best interests chapter updates the reader on the plethora of Court of Protection case law that has emerged and how the person's wishes have become central to the process.

An overview of Deprivation of Liberty Safeguards (DOLS) describes how issues with their use nationally provoked their reform and the subsequent development of Liberty Protection Safeguards (LPS), due to take the place of DOLS in autumn 2020. The chapter reviewing LPS provides a great starting point for understanding their practical use, although we await the associated Code of Practice to further flesh this out. Helpful chapters specifically address MCA use in the acute hospital (cleverly based on one sequential case study looking at realistic scenarios) and social care settings. My favourite chapter was the final one, which considers clinical ambiguities in the assessment of capacity which can often be clinically vexing, e.g. patient ambivalence, fluctuating capacity, unusual values/belief systems, self-harm, and consent and refusal of treatment.

This is a book for the jobbing clinician of all specialties, not just mental health. It provides a succinct yet comprehensive review that all healthcare professionals can benefit from. For students – both medical and nursing – this should be a required text. My only suggested improvement would have been to include a section on how the reader can access online MCA resources to help them keep abreast of developments. I hope the learned authors of this book continue to provide future editions as MCA case law evolves, including the inevitable future emergent case law, à la DOLS, from the introduction of LPS.

