# Mental Capacity Legislation edited by Rebecca Jacob, James Gunn, and Anthony Holland, Second Edition, RCPsych/Cambridge University Press, June 2019, Hardback, ISBN 9781108480369, £29.99 — CORRIGENDUM

**DOI:** 10.1192/bjb.2020.104

**Published:** 2022-06

**Authors:** Martin Curtice

This book review has an error in the title: *Mental Capacity Legislation* was in fact edited by Rebecca Jacob, Michael Gunn and Anthony Holland. We apologise for the error.
